# Oscillating primary transcripts harbor miRNAs with circadian functions

**DOI:** 10.1038/srep21598

**Published:** 2016-02-22

**Authors:** Haifang Wang, Zenghua Fan, Meng Zhao, Juan Li, Minghua Lu, Wei Liu, Hao Ying, Mofang Liu, Jun Yan

**Affiliations:** 1 CAS-MPG Partner Institute for Computational Biology, Shanghai 200031, China; 2 Institute of Neuroscience, CAS Center for Excellence in Brain Science and Intelligence Technology, Shanghai 200031, China; 3 Institute for Biochemistry and Cell Biology, Shanghai 200031, China; 4 Institute for Nutritional Sciences, Shanghai Institutes for Biological Sciences, Chinese Academy of Sciences, Shanghai 200031, China; 5 University of Chinese Academy of Sciences, Shanghai 200031, China

## Abstract

The roles of miRNAs as important post-transcriptional regulators in the circadian clock have been suggested in several studies. But the search for circadian miRNAs has led to disparate results. Here we demonstrated that at least 57 miRNA primary transcripts are rhythmically transcribed in mouse liver. Most of these transcripts are under the regulation of circadian transcription factors such as BMAL1/CLOCK and REV-ERBα/β. However, the mature miRNAs derived from these transcripts are either not oscillating or oscillating at low amplitudes, which could explain the inconsistency of different circadian miRNA studies. In order to show that these circadian primary transcripts can give rise to miRNAs with circadian functions, we over-expressed one of them, miR-378, in mouse by adenovirus injection. We found a significant over-representation of circadian oscillating genes under-expressed by miR-378 over-expression in liver. In particular, we observed that miR-378 modulates the oscillation amplitudes of *Cdkn1a* in the control of cell cycle and *Por* in the regulation of oxidation reduction by forming partnership with different circadian transcription factors. Our study suggests that circadian transcription of miRNA at primary transcript level can be a good indicator for circadian miRNA functions.

The daily changes in physiology and behaviors can be observed in almost all organisms. In mammals, a master clock located in the suprachiasmatic nucleus (SCN) of the hypothalamus drives circadian rhythms, adjusts itself according to the light input from the eyes, and synchronizes clocks in peripheral tissues such as the liver and kidney. At the molecular level, it has been shown that the negative transcriptional–translational feedback loops formed by a set of key circadian regulators (BMAL1, CLOCK, PER1, PER2, PER3, CRY1, CRY2, REV-ERBα, REV-ERBβ) are responsible for giving rise to the circadian physiology^1^,^2^. It is now known that transcriptional regulation is not the only way of circadian control. Comparing the nascent and mature mRNA transcripts on the whole genome level in mouse liver, Menet *et al.* claimed that only 28.4% of the rhythmic mRNAs are accompanied by rhythmic transcription^3^. Recently published circadian proteomic data showed that as much as 50% of the circadian oscillating proteins are derived from non-rhythmic mRNAs^4^,^5^. These observations strongly suggest that post-transcriptional regulation plays a substantial role in modulating temporal gene expression for proper circadian function.

MicroRNAs (miRNAs) are small non-coding RNA molecules that can regulate the expression of target genes by mRNA degradation or translational repression^6^,^7^. The roles of miRNAs in the circadian clock have been suggested in several organisms including fruit fly, mouse, human, and chicken^8^. In mammals, two brain specific miRNAs, miR-219 and miR-132, regulated by BMAL1/CLOCK and CREB proteins respectively, affect the circadian period and are involved in light dependent resetting of the clock in SCN^9^. MiR-192/194 cluster could inhibit the expression of *Per* family genes in HeLa cells^10^. MiR-142-3p directly targets *Bmal1* and is in turn regulated by BMAL1/CLOCK in the blood stream of human^11^.

During miRNA biogenesis, miRNAs are first transcribed by RNA polymerase II (Pol II) in the nucleus from DNA into primary transcripts, which are subsequently cleaved by Drosha and Pasha into miRNA precursors of around 80 nt. Then the miRNA precursors were translocated into the cytosol by RAN-GTP, Exportin-5 and processed into mature miRNAs^6^. To date, there have been several studies using genome-wide profiling experiments to identify the circadian miRNAs at the mature level. Xu *et al.* identified a sensory organ-specific miRNA cluster including miR-96, miR-182 and miR-183, oscillating in the mouse retina in a circadian manner^12^. By microarray-based expression profiling of both miRNA and mRNA in mouse liver, Na *et al.* identified 85 liver circadian miRNAs^13^. Vollmers *et al.* identified 30 mouse liver circadian miRNAs by miRNA-sequencing (miR-seq)^14^. However, the overlapped circadian miRNAs between the two high-throughput profiling studies in mouse liver are limited. These discrepancies raised the question about the approach to identify circadian miRNAs from their circadian expression. Gatfield *et al.* revealed that a liver specific miRNA, miR-122 regulated by REV-ERBα/β, shows strong circadian oscillation at the primary transcript level but not at the mature level. Nevertheless, they demonstrated that miR-122 can influence the circadian accumulation of their targets and plays an important role in circadian regulation of metabolism in liver^15^. Later, it was shown that miR-122 can also modulate the rhythmic expression of *Nocturnin*, which is a circadian clock-regulated deadenylase and is important for post-transcriptional control in circadian rhythm^16^. Using MEF cells derived from DICER knockout mice, Chen *et al.* showed that miRNAs including miR-24, miR-29a, and miR-30a are required for generating a time delay for the circadian oscillator although these miRNAs themselves do not show circadian expression^17^. Recently, Du *et al.* showed that the binding sites of miR-24 on the 3′ Untranslated Region (3′UTR) of *Per1 and Per2* are important for *Per1 and Per2*′s circadian expression in DICER liver conditional knockout (cKO) mice^18^. These studies suggest that the circadian oscillations of miRNAs at mature level may not be necessary for miRNA’s circadian functions.

In this study, we embarked a different approach to systematically investigate the circadian expression of primary transcripts of miRNAs in mouse liver. By integrating recently published circadian Nascent-sequencing (Nascent-seq) and global run-on sequencing (GRO-seq) data in mouse liver, we first identified 57 circadian primary transcripts of miRNAs exhibiting consistent circadian oscillations across the two datasets, including miR-122, miR-24 and miR-29a with known circadian functions. Next by integrating ChIP-seq data of circadian regulators, we found that most of these primary transcripts are under the regulation of core circadian regulators. Comparing with the sequencing data of mature miRNAs, we only identified four miRNAs showed consistent circadian phases and periods between primary and mature miRNA transcripts. We observed that the amplitudes of circadian oscillations of mature miRNAs were much lower than those of the miRNA primary transcripts most likely due to the long half-lives of miRNAs. The absence of high amplitude circadian oscillations of miRNAs at mature level can explain the inconsistency between various circadian miRNA datasets. To explore the circadian functions of the miRNAs derived from these circadian primary transcripts, we focused on miR-378, a miRNA without known circadian functions, and examined the transcriptome changes in mouse livers upon miR-378 over-expression at two circadian time points, CT10 and CT22. First, we observed a significant over-representation of the circadian oscillating genes affected by miR-378 over-expression. Cell cycle genes including *p21* (*Cdkn1a*) were further enriched in the affected circadian oscillating genes, which implied that miR-378 could mediates the circadian control of cell cycle. Our examination of circadian regulatory network involving miR-378 suggests that miR-378 mediates the circadian control of cell cycle and metabolism by forming partnership with circadian transcription factors (TFs). Taken together, our study shows that circadian oscillations of miRNAs at primary transcript level rather than mature level can give rise to important circadian functions.

## Results

### 57 miRNA primary transcripts are circadianly transcribed in mouse liver

**Figure 1:**
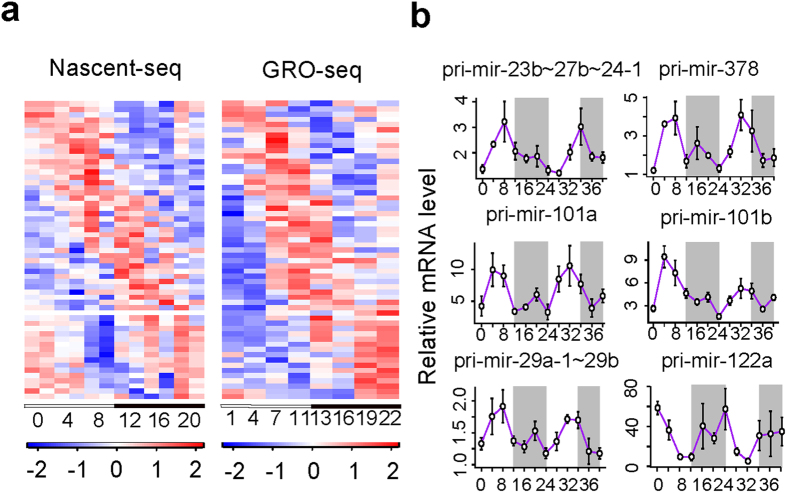
Circadian expression of miRNA primary transcripts. (**a**) Two heatmaps showed the expression patterns of circadian miRNA primary transcripts from Nascent-seq and GRO-seq respectively. High (red) and Low (blue) expression values as Z-scored normalized ratios are indicated in the color scale bar at the bottom. Black/white bars indicate the circadian day (white) and night (black) of the consecutive circadian cycles. (**b**) Expression patterns of circadian miRNA primary transcripts validated by qPCR. The x-axis represents the circadian time (CT), while the y-axis represents the relative expression values normalized by those of *Actb*. The white regions represent subjective day while the gray regions represent subjective night.

To systematically identify circadian primary transcripts of miRNAs, we integrated the recently published data of Nascent-seq^3^ and GRO-seq^19^ of mouse livers collected in circadian cycles. First, we used Vespucci program^20^ to assemble the primary miRNA transcripts in mouse liver *de novo* from strand-specific mouse liver circadian GRO-seq data^19^ (see materials and methods). Out of the 1186 mouse miRNAs with known precursor sequences (mirbase release 21), we identified 558 miRNA primary transcripts expressed in mouse liver corresponding to 611 miRNA precursor sequences (Supplementary Table S1). In comparison, Saini *et al.* only identified 39 mouse intergenic miRNA primary transcripts based on the transcription features including CpG island, 5′ CAGE, transcription start site (TSS) and poly-A signals as well as public cDNA and EST sequences in mouse^21^. Among their nine primary transcripts overlapped with our result, our approach has recovered longer primary transcripts with extended 5′ and/or 3′ ends (Supplementary Fig. S1 and Supplementary Table S2). We then mapped the sequencing reads from GRO-seq and Nascent-seq onto our assembled primary transcripts by Bowtie program^22^. We quantified the expression levels of miRNA primary transcripts and identified those showing significant circadian oscillations consistently from both GRO-seq and Nascent-seq by fitting the expression values to cosine functions with shifting phases respectively (materials and methods). 57 miRNA primary transcripts corresponding to 60 miRNA precursors were defined as circadian miRNA primary transcripts (Table 1 and Fig. 1a). Six of them were selected to be validated by qPCR using specific primers. In our independently collected circadian mouse liver samples, all six miRNA primary transcripts exhibited significant circadian oscillations and consistent circadian phases with the two high-throughput sequencing datasets (Fig. 1b). Also consistent with Gatfield *et al.*’s result^15^, the primary transcript of miR-122 showed significant circadian oscillation with a peak around CT0 and a trough at CT12. Several other miRNAs that have been previously implicated in circadian regulation were also identified in our result. These include miR-24 and miR-29a that show no circadian expression at mature levels but were reported to be involved in the regulation of circadian period^17^.

### Circadian regulation of oscillating miRNA primary transcripts

**Figure 2:**
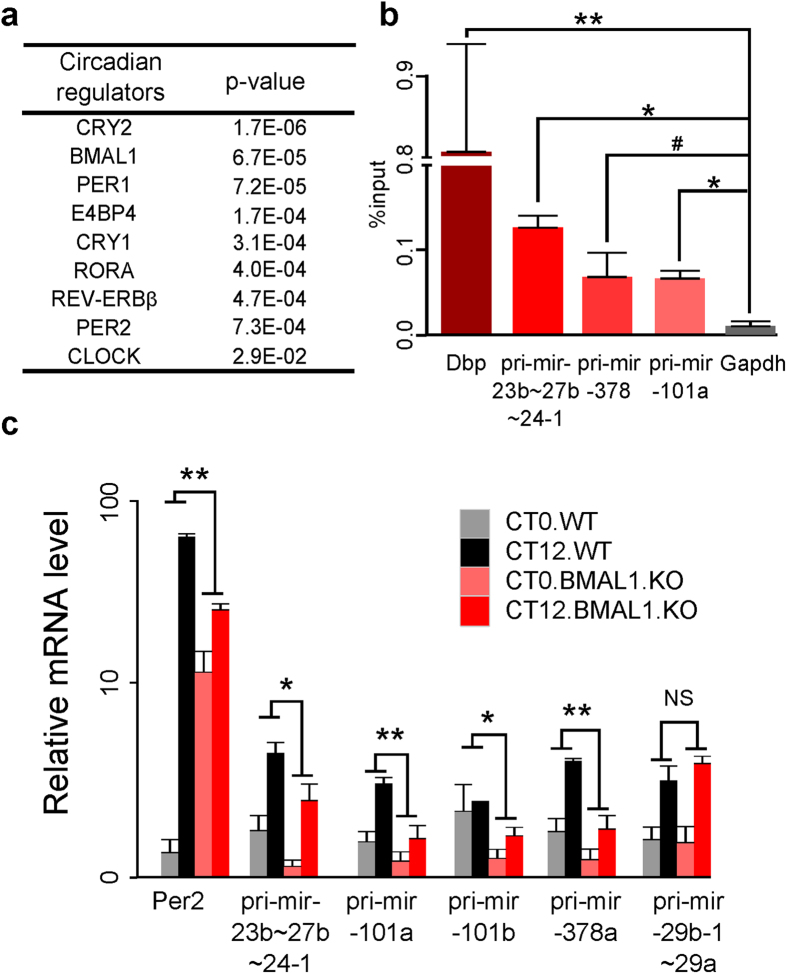
The regulation of circadian miRNA primary transcripts by circadian regulators. (**a**) circadian regulator binging sites are enriched on the promoters of the circadian oscillating miRNA primary transcripts comparing to all miRNA primary transcripts. Enrichment p-values were calculated by proportion test. (**b**) BMAL1 ChIP-PCR showed that the promoter regions of pri-mir-23b~27b~24-1, pri-mir-378, and pri-mir-101a are bound by BMAL1/CLOCK. *Gapdh* is used as the negative control, while Dbp is used as the positive control. **represents Student’s t-test (unpaired, two-sided) p-value < 0.01, *represents Student’s t-test (unpaired, two-sided) p-value < 0.05. #represents Student’s t-test (unpaired, two-sided) p-value < 0.1 (marginally significant). (**c**) qPCR analysis of circadian miRNA primary transcripts of liver-specific *Bmal1* cKO mice and wild-type mice. *Per2* is used as the positive control. The y-axis has an exponential scale. Two-way ANOVA using circadian time (CT0 and CT12) and genotype (*Bmal1* cKO vs. wild-type) as two factors were applied to assess the statistical significance. **represents ANOVA p-value for genotype < 0.01, *represents ANOVA p-value for genotype < 0.05, NS stands for not significant p-value.

To investigate if the oscillating miRNA primary transcripts are under the control of circadian TFs, we conducted the promoter analysis for circadian miRNA primary transcripts. First, we confirmed that our *de novo* assembled primary miRNA transcripts indeed captured the 5′ ends of the transcripts by comparing their 5′ ends with the mouse liver specific promoter marker (H3K4me3) and Pol II marks. For 42 out of 57 circadian miRNA primary transcripts, we can find both H3K4me3 and Pol II marks on the 5′ ends of our assembled primary miRNA transcripts. For seven of them, we can only find H3K4me3 marks. This indicates that the 5′ ends of our assembled primary transcripts extended to the *bona fide* promoter regions of miRNAs. For the eight transcripts that do not overlap with H3K4me3 and Pol II marks, we corrected the 5′ ends of transcripts to the centers of the nearest H3K4me3 marks. Previously, Figueredo *et al.* have tried to identify the transcriptional regulators of the circadian miRNAs just by TF binding site prediction based on position weight matrix (PWM)^23^. However, the PWM-based TF binding site prediction tends to have high false positive and false negative rates. In the recent years, the ChIP-seq data of the core circadian regulators (BMAL1, CLOCK, PER1, PER2, CRY1, CRY2, REV-ERBα, REV-ERBβ, RORA, E4BP4) have been published in mouse liver^3^,^19^,^24^,^25^,^26^,^27^. By searching for circadian regulator binding sites on the promoters of circadian miRNA primary transcripts based on these ChIP-seq data, we identified potential circadian regulators of these miRNA primary transcripts (Supplementary Table S5). Comparing to all miRNA primary transcripts, we found that the promoters of circadian miRNA primary transcripts are more likely to be bound by most of the circadian regulators (Fig. 2a) and all promoters of the circadian miRNA primary transcripts, except for pri-mir-6971, contain at least one core circadian regulator binding site (Supplementary Table S5). Our BMAL1 ChIP-PCR experiment in mouse livers validated the physical binding of BMAL1 on the promoters of three selected circadian miRNA primary transcripts, pri-mir-23b~27b~24-1, pri-mir-101a, and pri-mir-378 (Fig. 2b). To further test if the regulatory relationships inferred from circadian TF ChIP-seq data are functional, we examined the expression changes of the circadian miRNA primary transcripts between liver-specific *Bmal1* cKO mice and wild-type mice by qPCR. We observed that four out of five tested BMAL1 regulated circadian miRNA primary transcripts containing BMAL1 binding sites are significantly under-expressed in liver-specific *Bmal1* cKO mice compared to wild-type mice at CT12 (Fig. 2c). It has been reported that miR-24 regulates *Per1* and *Per2* expression and is required for generating a time delay for the circadian oscillator^17^. Here we showed that miR-24 primary transcript is regulated by BMAL1/CLOCK. In summary, our results indicated that circadian miRNA primary transcripts are indeed regulated by circadian clock through core circadian TFs in mouse liver.

### Dampening of circadian oscillation of miRNA expression at mature level

**Figure 3:**
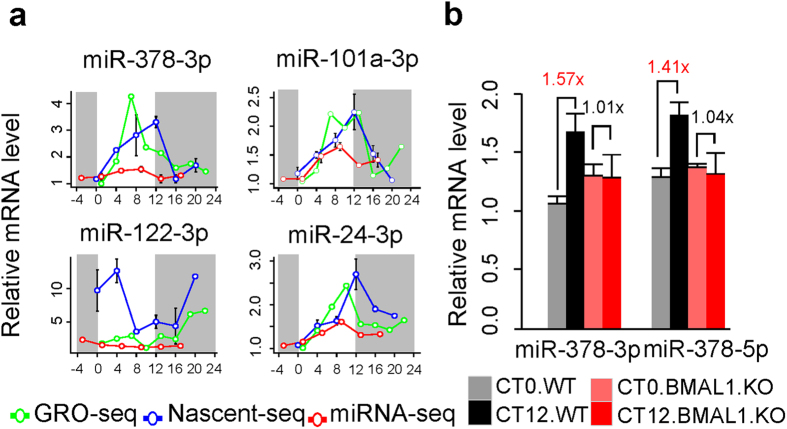
Circadian expression of mature miRNAs. (**a**) Comparison of the expression of circadian miRNA primary transcripts and their corresponding miRNAs at mature level. The green lines represent the expression values of primary transcripts from GRO-seq. The blue lines represent the expression values of primary transcripts from Nascent-seq. The red lines represent the expression values of mature transcripts from miRNA-seq. The white regions represent subjective day while the gray regions represent subjective night. (**b**) Expression patterns of miR-378-3p and miR-378-5p in liver-specific *Bmal1* cKO mice and wild-type mice at CT0 and CT12. Student’s t-test (unpaired, two-sided) p-value < 0.05 are highlighted in red color for comparisons between liver-specific *Bmal1* cKO mice with wild-type mice.

To date, two high-throughput studies have examined circadian expression of miRNAs at the mature level including miRNA microarray data study by Na *et al.*^13^ and miRNA-seq study by Vollmers *et al.*^14^. These two studies reported 85 and 30 circadian mature miRNAs respectively in mouse liver. Surprisingly, only two miRNAs (miR-26b and miR-150) appeared in common between both circadian miRNA lists. Although we were unable to obtain the raw data from Na *et al.*, a detailed examination of the original expression profiles of miR-26b and miR-150 in Vollmers *et al.*’s data suggested that miR-26b, i.e. miR-26b-5p, does not appear to be circadian oscillating (Supplementary Fig. S2a). Only miR-150-5p showed a moderate circadian oscillation in Panda’s data with a peak time around CT8, which is close to that reported in Na *et al.*’s data (Supplementary Fig. S2b). This lack of consistency between the two circadian miRNA studies at mature level is in stark contrast to the overall consistency of circadian primary transcripts based on Nascent-seq and GRO-seq data. To investigate if circadian primary transcripts give rise to miRNAs with circadian expression at mature level at all, we re-analyzed the circadian miRNA-seq data from Vollmers *et al.*’ using relaxed criteria (one-way ANOVA p-value < 0.05 and cosine fitting p-value < 0.05) (materials and methods). We found that four miRNAs (miR-24-3p, miR-101a-3p, miR-378-3p and miR-122-3p) showed significant circadian oscillations at mature levels with peak times close to those of their primary transcripts. However, the relative amplitudes of circadian oscillation at mature level are around 2-3 folds lower than those in primary transcripts (Fig. 3a). We next investigated whether mature miRNA expression are also regulated by BMAL1/CLOCK. We examined the expression changes of mature miR-378-3p and miR-378-5p in liver-specific *Bmal1* cKO mice and wild-type mice by miRNA specific qPCR (materials and methods). We found that the weak oscillations of miR-378-3p as well as miR-378-5p were abolished in liver-specific *Bmal1* cKO mice (Fig. 3b). These observations suggested that miR-378 is under the regulation of BMAL1/CLOCK at both primary and mature levels.

### Genome-wide effects of miR-378 over-expression

In spite of weak or undetectable circadian expression of mature miRNAs, we found that at least 57 circadian miRNA primary transcripts including pri-mir-122 and pri-mir-24 do show strong circadian expression and are under circadian regulation. As miR-122 and miR-24 have been previously reported to be involved in circadian rhythm, we wondered if other circadian miRNA primary transcripts also harbor miRNAs with important circadian functions. To address this question, we selected miR-378 derived from pri-miR-378 transcript for further investigation. The expression of miR-378 shows circadian oscillation peaking at CT10 and is regulated by BMAL1/CLOCK. While miR-378 has been suggested to be related to cell cycle, cell proliferation and transformation, metabolism, adipocyte differentiation and stress responses^28^,^29^,^30^,^31^, the functions of miR-378 in circadian rhythm have not yet been reported. To explore the circadian functions of miR-378, we injected adenovirus over-expressing both miR-378-3p and miR-378-5p into mice (termed Ad-378) while the control mice were injected with null virus (termed Ad-null, materials and methods). The efficiency of miR-378 over-expression in liver was about 50 folds as examined by miRNA specific qPCR (Supplementary Fig. S3). The liver samples of Ad-378 and Ad-null mice were collected at CT10 and CT22 corresponding to the expression peak and trough of miR-378 respectively. The global gene expression profiles were measured by RNA-sequencing (RNA-seq). We applied two-way ANOVA using circadian time (CT10 and CT22) and types of treatment (Ad-378 vs. Ad-null) as two factors to identify the genes that are affected by miR-378 over-expression.

**Figure 4:**
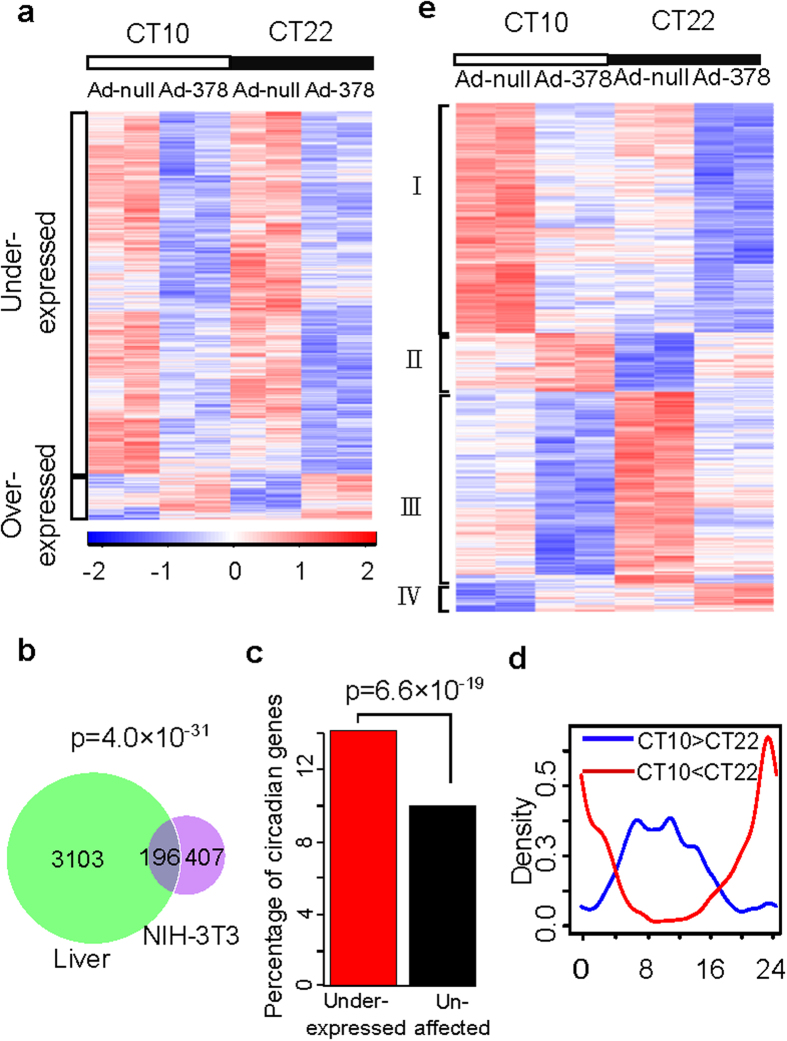
Transcriptome profile of miR-378 over-expression in mouse liver. (**a**) Heatmap of all genes affected by miR-378 over-expression. The genes are clustered into two groups based on their expression patterns. High (red) and low (yellow) expression values as Z-scored normalized ratios are indicated in the color scale bar at the bottom. Black/white bars indicate the circadian day (white) and night (black) of the consecutive circadian cycles. (**b**) The number of overlapping genes between under-expressed genes in Ad-378 comparing to Ad-null (green circle) and miR-378-3p targets from Ruckerl *et al.*’s data (purple circle). Enrichment p-value was determined by Fisher’s exact test. (**c**) Enrichment of circadian transcripts in under-expressed genes upon miR-378 over-expression. Enrichment p-value was determined by proportion test. (**d**) Comparison of the circadian peak times in our data with the peak times from mouse liver circadian database. The red curve represents the circadian peak time distribution for genes over-expressed at CT22 in our data. The blue curve represents the circadian peak time distribution for genes over-expressed at CT10 in our data. (**e**) Heatmap of the circadian oscillating genes affected by miR-378 over-expression. The transcripts are clustered into four groups based on their expression patterns. High (red) and low (yellow) expression values as Z-scored normalized ratios are indicated in the color scale bar at the bottom. Black/white bars indicate the circadian day (white) and night (black) of the consecutive circadian cycles.

Using ANOVA p-value for the factor of treatment, i.e. Ad-378 vs. Ad-null, less than 0.05 as the cutoff, we obtained 3,718 genes affected by miR-378 over-expression (Fig. 4a). Among these genes, the under-expressed genes in Ad-378 compared to Ad-null were over-represented by as much as eight-fold, consistent with the inhibitory role of miR-378 in gene regulation. Functional annotations of these genes showed that the most enriched function for the under-expressed genes is cell cycle (DAVID enrichment p-value = 2.6×10^−44^, using all mouse genes as the background). This is also consistent with the known function of miR-378 in cell cycle. Integrating with the miRNA-target interaction data based on both computational prediction and large-scale AGO CLIP-sequencing data from starBase (http://starbase.sysu.edu.cn/), we found a significant enrichment of the putative miR-378 targets in the under-expressed genes (Fisher’s exact test p-value = 1.43×10^−12^, odd ratio = 1.82). Comparing with miR-378 target genes identified by another genome-wide study of miR-378-3p in NIH-3T3 cells^32^, we again found a significant overlap between their result and our under-expressed genes by miR-378 over-expression in mouse liver (Fig. 4b).

### Circadian oscillating genes are significantly affected by miR-378 over-expression

We observed that the under-expressed genes were significantly enriched for liver circadian oscillating genes complied from six published circadian microarray datasets in mouse liver (mouse liver circadian database, materials and methods). Namely, a significantly elevated proportion (14.2%) of the under-expressed genes are circadian oscillating in mouse liver as compared to only 10% of the un-affected genes upon miR-378 over-expression (Fig. 4c). Using the p-value for the circadian time factor less than 0.05 as the cutoff, 2,266 genes showing significant differences in expression between CT10 and CT22 were identified. Comparing with the circadian peak time data from our mouse liver circadian database (materials and methods), the genes over-expressed at CT10 compared to CT22 (CT10 > CT22) in our study predominantly have circadian peak times around CT10 in published data while the genes over-expressed at CT22 compared to CT10 (CT10 < CT22) in our study have circadian peak times around CT20 (Fig. 4d). The core circadian genes (*Bmal1, Clock, Rev-ervα, Rev-ervβ, Per1, Per2, Per, Cry1, Cry2, and Cry3*) all showed significant differences in expression between CT10 and CT22 in our result, which is consistent with the literatures (Supplementary Fig. S4).

Furthermore, 833 genes showing significant difference in expression between CT10 and CT22 were affected by miR-378 over-expression (ANOVA p-value for treatment < 0.05 and ANOVA p-value for time < 0.05) (Fig. 4e). These include at least one core circadian gene, *Cry2* (Supplementary Fig. S4). These genes were clustered into four groups based on the hierarchical clustering of their gene expression. Group I genes peaked around CT10 and were under-expressed in Ad-378 (377 genes). Group II genes peaked around CT10 and were over-expressed in Ad-378 (96 genes). Group III genes peaked around CT22 and were under-expressed in Ad-378 (314 genes). Group IV genes peaked around CT22 and were over-expressed in Ad-378 (46 genes). Since the under-expressed genes are more likely to be the direct targets of miR-378, we then focused on the genes in Group I and Group III and defined the genes in these two groups as miR-378 directly targeted circadian oscillating genes, denoted as miR-378 circadian targets. We applied DAVID program^33^ to identify the enriched biological functions for each group of genes using all mouse genes as the background. Interestingly, we found that the most significantly enriched function for Group I is cell cycle while the most significantly enriched function for Group III is positive regulation of programmed cell death and apoptosis (Supplementary Table S9). Five miR-378 circadian targets involved in cell cycle regulation (Ccne1, Runx3, Cdkn1a, Bbc3, and Bcl2) were selected to be validated by qPCR. Consistent with the RNA-seq data, all of them showed significant changes upon miR-378 over-expression. For all of them we can find the putative miR-378 binding sites on their 3′UTRs using Miranda algorithm (Fig. S5). miR-378 has been mostly described as an oncogene-like miRNA in different cancer cell lines^34^,^35^ and was reported to promote tumor growth, cell survival, and cell transformation^36^,^37^. We observed that both cyclin E1 (*Ccne1*) peaking around CT10, which is required for cell cycle G1/S transition and *Cdkn1a* peaking around CT22, which inhibits G1/S transition, are miR-378 circadian targets. It suggests that miR-378 may have dual roles in the circadian regulation of cell cycle progression.

### miR-378 is involved in circadian gene regulation

**Figure 5:**
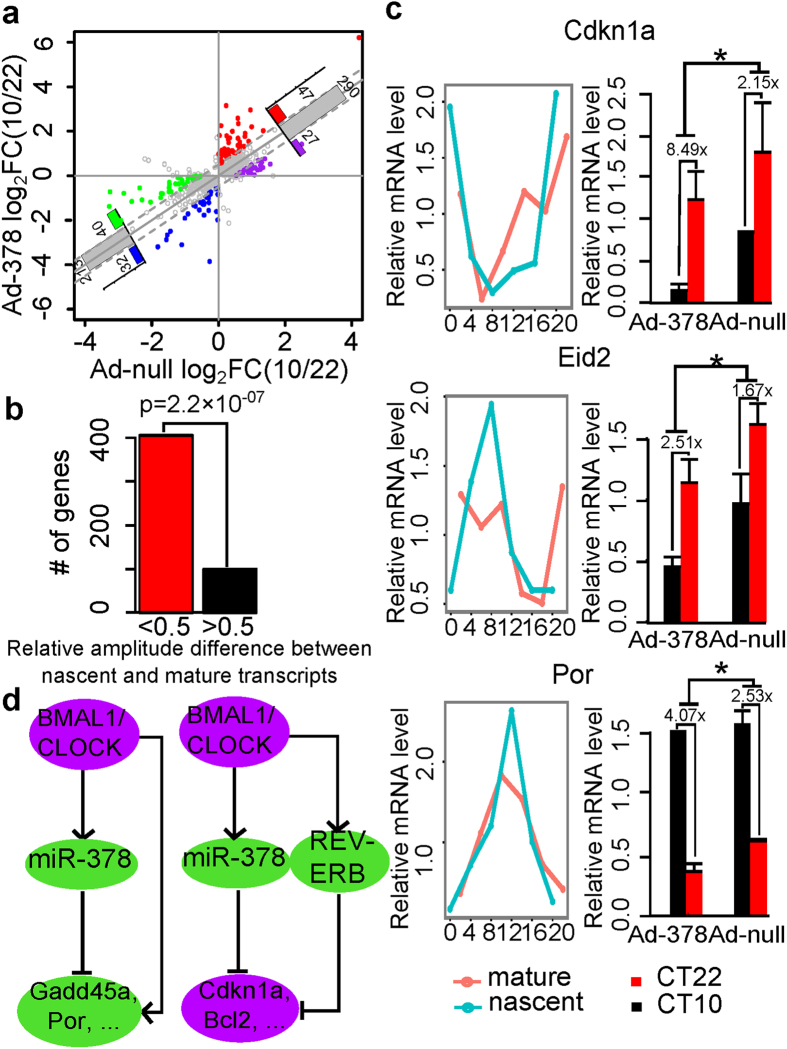
miR-378 in circadian gene regulation. (**a**) Comparison of the log2-transformed fold changes of CT10 and CT22 (log_2_FC(CT10/CT22)) between Ad-null and Ad-378 of miR-378 circadian targets (Group I and Group III genes in Fig. 4e). The solid grey line represents y = x. The dashed grey lines represent y = x ± 0.5. The colored dots represent the genes with log_2_FC(CT10/CT22) differences between Ad-378 and Ad-null larger than 0.5, while the grey nodes represent the genes with the difference less than 0.5. Number of genes in each group is indicated in the bar plot. (**b**) Comparison of the relative amplitudes (log2-transformed peak to trough ratio) between nascent and mature transcripts of the grey dots in 5a. Red bar represents the genes that do not show relative amplitude changes (difference of the relative amplitude less than 0.5). Black bar represents the genes with relative amplitude changes (difference of the relative amplitude larger than 0.5). Enrichment p-values were calculated by Fisher’s exact test using the relative amplitude differences between nascent and mature transcripts of all mouse genes as the background. (**c**) The expression patterns of the three genes (*Cdkn1a*, *Eid2*, and *Por*) that showed increased relative amplitudes in nascent transcript comparing to mature transcript and in Ad-378 comparing to Ad-null. The left figures show the expression patterns of nascent and mature transcripts and the right figures show the expression patterns upon miR-378 over-expression. *represents ANOVA p-value for treatment < 0.05. (**d**) Two gene regulatory motifs formed by miR-378 and circadian TFs (BMAL1/CLOCK, REV-ERBα/β). The green nodes represent the genes peaking around CT10, while the purple nodes represent the genes peaking around CT22.

For the mRNA transcripts under circadian transcriptional regulation, the inclusion of miRNA regulation will affect their mRNA degradation and influence their baseline levels as well as their amplitudes of circadian oscillations. Comparing the relative amplitude defined as the log2-transformed fold changes between CT10 and CT22, i.e. log_2_FC(CT10/CT22), of Ad-378 and Ad-null, we observed that 80% of miR-378 circadian targets showed little or no differences in relative amplitudes (Fig. 5a), i.e. relative amplitude differences less than 0.5. These genes also tend to show little or no differences in their relative amplitudes between their nascent transcripts measured in Nascent-seq and mature mRNA transcripts measured in RNA-seq (Fig. 5b). This suggests that these genes may be already under strong regulation by miR-378 such that the over-expression of miR-378 does not further increase their circadian amplitudes. However we still identified 47 circadian oscillation genes peaking around CT10 (red dots in Fig. 5a) and 32 genes peaking around CT22 (blue dots in Fig. 5a) showing increased relative amplitudes upon miR-378 over-expression. Cell cycle related genes are significantly enriched among those genes (DAVID enrichment p-value = 3.3×10^−5^, using all mouse genes as the background). In particular, *Cdkn1a* showed more than four-fold relative amplitude increase upon miR-378 over-expression. Therefore, miR-378 can influence the relative circadian amplitudes of cell cycle related genes by regulating their mRNA degradation rates. For these genes, we reasoned that the relative amplitudes of their premature transcripts may be also higher than those of their mature transcripts. Indeed, we identified that three genes (*Cdkn1a*, *Eid2*, and *Por*) showed significant increased relative amplitudes in their nascent transcripts comparing to the mature transcripts in a similar fashion as in Ad-378 comparing to Ad-null (Fig. 5c).

We next examined the potential cooperation between core circadian TFs and miR-378 in circadian gene regulation. It has been known that the circadian oscillating genes peaking around CT10 are more likely regulated by BMAL1/CLOCK while the genes peaking around CT22 are more likely regulated by REV-ERBα/β^19^. Therefore, we overlapped the Group I genes with the BMAL1/CLOCK regulated genes and Group III genes with the REV-ERBα/β regulated genes. Here we defined BMAL1/CLOCK and REV-ERBα/β regulated genes by both physical TF binding data from ChIP-seq and functional data from TF knock-out RNA-seq or microarray from ours and published data (materials and methods). As such, we constructed a gene network involving miR-378 and circadian TFs (Fig. S7). We observed two types of regulatory motifs (Fig. 5d). In the first motif, which is a feed-forward loop (FFL), miR-378 cooperated with BMAL1/CLOCK to regulate the genes including *Gadd45a* and *Por* with the circadian peak around CT10. In the second motif, miR-378 cooperated with REV-ERBα/β to regulate the genes including *Cdkn1a* and *Bcl2* with circadian peaks around CT22.

For the miR-378 circadian target genes that lack circadian transcriptional regulation, miR-378 alone can lead to their circadian expression by rhythmic post-transcriptional regulation. Lück *et al.* have previously identified 493 circadian oscillating genes under rhythmic post-transcriptional regulation^38^. We overlapped the miR-378 circadian targets in our study with Lück *et al.*’s result. We found that there was a significant overlap between these two gene sets (Fisher’s exact test p-value = 4.74×10^−6^, odd ratio = 2.65). Interestingly, five genes involved in the regulation of transcription (*Rdbp, Elp3, Fus, Irf2, Pnrc2*) were among the overlapped genes. These genes either showed significant increases in oscillation amplitudes of mature transcripts comparing to nascent transcripts such as *Fus* or large phase shift between nascent and mature transcripts such as *Rdbp* and *Pnrc2*. Meanwhile, they were also significantly under-expressed in Ad-378 vs. Ad-null in our study (Supplementary Fig. S6). *Fus* is a circadian oscillating gene that shows consistent circadian expression across many different tissues in mouse^39^. Consistent with our result, Ma *et al.* showed that miR-378 promotes the migration and metastasis of liver cancer cells by down-regulating *Fus* expression^29^. Therefore, circadian post-transcriptional regulation of *Fus* by miR-378 can further contribute to the circadian regulation of cell cycle. The post-transcriptional regulation of these genes by miR-378 may play a predominant role in their circadian regulation.

## Discussion

In mouse, liver is the most extensively studied peripheral tissue in circadian rhythm so far. Various high-throughput technologies including microarray, RNA-seq, and ChIP-seq have been applied to the study of circadian rhythm in mouse liver. Several papers have reported the functions of miRNAs in circadian rhythm in mouse liver. But the studies on specific miRNAs showed that the miRNAs of circadian functions are either arrhythmic or only rhythmic^17^ at primary transcript level^15^, while two systematic investigations of circadian miRNAs in mouse liver at mature level resulted in quite different miRNA lists^13^,^14^. In this study, we first identified 57 miRNA primary transcripts showing significant and consistent circadian oscillations by integrating two independent next-generation sequencing data of nascent transcripts in mouse liver. We investigated their regulations by core circadian regulators by integrating the circadian ChIP-seq data. Notably, three of them have already been reported to be involved in circadian regulation, which suggests that the miRNAs rhythmic at primary level could harbor circadian functions. We then compared the primary miRNA expression with the mature miRNA expression and found only four miRNAs that showed consistent circadian phases with their primary transcripts while the circadian amplitudes of the mature miRNAs are only around one third of the primary transcripts. The average half-life of mature miRNAs in mammals was estimated to be around 119h^40^. Under such long half-lives, most circadian primary transcripts will not lead to miRNAs with detectable circadian oscillation at mature level, which may also explain the inconsistency of the high-throughput studies designed to identify the mouse liver circadian miRNAs. Therefore, we proposed that the circadian expression of miRNAs at primary level can be a better criterion to define circadian miRNAs.

In the light of our result, there are two scenarios of how miR-378 could exert its impact on circadian expression. First, Gatfield *et al.*^15^ suggested that miRNAs such as miR-122 that are arrhythmic at mature level could contribute to the large amplitudes of oscillation of target mRNA transcripts by maintaining their high but constant degradation rates. Supporting this model, we identified 79 miR-378 circadian targets that showed increased relative amplitudes upon miR-378 over-expression. For the rest of miR-378 circadian targets that do not show significant amplitude changes, it is still possible that they are strongly regulated by miR-378 in wild type mice so that miR-378 over-expression can not increase their amplitudes further. This model can explain how the miRNAs that are either weakly rhythmic or arrhythmic at mature level could contribute to the circadian regulation. Second, we observed that the genes under rhythmic post-transcriptional regulation significantly enriched in miR-378 circadian targets. As the mature miRNA-378-3p and miRNA-378-5p still showed low amplitudes of circadian oscillation, the circadian expression of these targets may be driven by rhythmic post-transcriptional regulation through miRNA-378. In this scenario, miRNAs rhythmically regulate the degradation of the target mRNAs and thus are crucial for their circadian expression.

Circadian regulation is an essential control mechanism of cell cycle^41^. Our functional annotation has pointed to the cross talk between circadian rhythm and cell cycle through miR-378. Among the cell cycle genes targeted by miR-378, *Ccne1* peaking around CT10 is required for cell cycle G1/S transition, while *Cdkn1a*, an inhibitor of G1/S transition, shows circadian peak around CT22 and has long been shown to be repressed by REV-ERBα^42^. In NIH-3T3 cells, *Cdkn1a* is also significantly under-expressed in miR-378 over-expression and up-regulated in miR-378 inhibition^32^. We suggested that miRNA-378 might regulate different groups of target genes by cooperating with different circadian TFs (Fig. 5d). It has been shown that one of our motifs constituting a FFL is particularly prevalent in the miRNA-mediated regulatory network^43^. Through both of our motifs, miR-378 can further fine-tune the circadian amplitudes of target genes by increasing their degradation rates. In addition, miR-378 has been reported to be involved in metabolic process. In human breast cancer cells, miR-378 induces metabolic shift by inhibiting the expression of two PGC1β partners, ERRγ and GABPA. This leads to the decrease of gene expression in oxidative phosphorylation and the increase in glycolysis^44^. In mouse liver, it has been shown that miR-378 targets p110*α* and controls glucose and lipid homeostasis by modulating hepatic insulin signaling^30^. In our study, two metabolic genes (*Pdk4* and *Por*) showing robust circadian expression in mouse liver are under-expressed upon miR-378 over-expression. *Pdk4* is involved in glucose metabolism while *Por* is involved in oxidative reduction. Our findings may provide the explanation of the time-of-day dependent function of miR-378 in the regulation of cell cycle and metabolism.

In summary, our study suggests a new way of searching for miRNAs with circadian functions, i.e. among the miRNAs showing circadian expression in their primary transcripts. We have provided evidences for the role of miR-378 in circadian regulation. Future studies will be necessary to explore the circadian functions of the rest of circadian primary miRNA transcripts.

## Material and Methods

### Assembly of miRNA primary transcripts

Vespucci program^20^ developed for the identification of *de novo* transcripts from GRO-sequencing data, was applied to assemble the mouse liver primary miRNA transcripts from strand-specific mouse liver circadian GRO-seq data^19^ with two modified parameters, DENSITY_MULTIPLER = 100 and MAX_EDGE = 1000. The primary transcripts containing known miRNA precursors were extracted. The identified mouse liver primary transcripts were listed in Supplementary Table S1.

### Identification of circadian miRNA primary transcript

Sequencing reads from GRO-seq^19^ and Nascent-seq^3^ were mapped to mouse genome (mm9) by Bowtie2 program (default parameter)^22^. The read number of each primary transcript was normalized to reads per kilobase per million tags (RPKM).We searched for the circadian oscillating miRNA primary transcripts that showed significant and consistent circadian oscillation on both GRO-seq and Nascent-seq data by fitting them to the common cosine functions with 24 hours’ period and shifting phases as described previously^39^. The circadian primary transcripts were defined as the summed log2-transformed cosine fitting p-value > 9 and circadian phase difference <4. False discovery rate (FDR) was calculated by randomly shuffling the gene 1000 times. The cutoffs that we used corresponded to an overall FDR = 29% as computed from random permutation.

### Animal preparation and sample collection

All mice were males aged 8–12 weeks and were maintained on a C57BL/6J background. The mice were housed under a 12-h light/12-h dark regimen with food and water available ad libitum for one week before being switched to complete darkness. The mice were switched to complete darkness two days before execution. CT0 is defined as the time when the lights are turned on. The animal handing protocols have been approved by Institutional Animal Care and Use Committee of Institute for Nutritional Sciences. All experimental procedures performed followed the guidelines of the Institutional Animal Care and Use Committee of Institute for Nutritional Sciences. Ad-378 and Ad-null mice were generated as previously described^30^. Basically, DNA fragments encoding miR-378 were introduced into pENTR/U6 vector under the control of the human U6 promoter. 5 × 10^8^ plaque-forming unit viruses were administered to four Ad-378 mice through tail vein injection for miR-378 over-expression. Control viruses were injected to four Ad-null mice. Two Ad-378 and two Ad-null mice were sacrificed at CT10 and CT22 respectively eight days after injection. To validate the circadian expression of miRNA primary transcripts, liver samples from wild-type mice were collected. Starting at CT0, three mice were killed every four hours for 48 hours. Livers of all mice were quickly dissected and snap-frozen in liquid nitrogen and stored at −80 °C.

RNA samples of livers from three liver-specific *Bmal1* cKO mice and three wild-type mice (Bmal1^*flox/flox*^) sacrificed at CT0 and CT12 respectively were provided by Dr. Yi Liu (Institute for Nutritional Sciences). Basically, Bmal1^*flox/flox*^ mice were obtained from Jackson’s Lab (stock no. 007668). These mice have loxP sites flanking exon 8 of the *Bmal1* gene. Then the *Bmal1*^flox/flox^ mice were bred to mice expressing CRE recombinase with liver specific promoter. The resulting offsprings have the exons encoding BMAL1 basic helix-loop-helix (bHLH) domain deleted in the liver.

### qPCR of miRNA primary transcripts

Total RNA was isolated from mouse liver by Trizol (Invitrogen). Total RNA quantities were measured by a Nanodrop spectrometer. RNA quality was assessed by an Agilent Bioanalyzer. 500 ng of total RNA was reverse transcribed using random hexamer and the Superscript II reverse transcriptase (Invitrogen). 5 μl of RT product (1:10 diluted) and SYBR Green I Master Mix (Roche) were used in qPCR on a LightCycler 480 (Roche). The specificity of PCR was checked by melting curve analysis. In every qPCR assay, *Actb* was used as the control for any significant bias of starting materials across samples. Specific primers for miRNA primary transcripts were listed in Supplementary Table S3. The same qPCR procedure was applied to validate the expression pattern of cell cycle genes upon miR-378 over-expression, and the primers for the cell cycle genes were listed in Supplementary Table S10.

### Promoter analysis of circadian miRNAs

Mouse liver H3k4me3 and PolII ChIP-seq data from Bing Ren’s lab^45^ were used to confirm that our *de novo* assembled primary miRNA transcripts indeed captured the 5′ end of the transcripts. The sequencing reads were mapped to mouse genome (mm9) using bowtie2 program (default parameter)^22^. MACS program^46^ was applied to identify the binding peaks of ChIP-seq data (MACS 1.4.2, default parameter). For the six transcripts that do not overlap with H3K4me3 and Pol II marks, we corrected their 5′ ends of transcripts to the centers of the nearest H3K4me3 or Pol II marks.

The fastq files containing the raw sequence reads of the circadian ChIP-seq data were downloaded from GEO database (http://www.ncbi.nlm.nih.gov/geo) and listed in Supplementary Table S4. For the ChIP-seq experiments conducted across multiple circadian time points, we selected the time point corresponding to the binding peak of each regulator. The reads were mapped to mouse mm9 genome using bowtie2 (default parameters)^22^ while bowtie1 were used for to Koike *et al.*’s color-space SOLID RNA-seq data. MACS program^46^ was applied to identify the binding sites of each regulator (MACS 1.4.2, default parameter). The regulators having binding sites between upstream 5,000 bp of 5′ end and 3′ end of the transcripts were defined as the regulators of the miRNA primary transcripts. For the six BMAL1 ChIP-seq datasets, we defined the physical binding of BMAL1 on the transcripts if the binging events appear in at least two datasets. For the other regulators, we defined their binding as long as their binding events appear in at least one dataset. The number of datasets supporting the binding of regulators on each miRNA primary transcript was listed in Supplementary Table S5.

BMAL1 ChIP-PCR analysis, analysis of mouse liver circadian miRNA-seq data, qPCR of mature miR-378-3p and miR-378-5p expression, circadian miRNA target identification, RNA-sequencing and data analysis, miR-378-3p targets in NIH-3T3 cell, mouse liver circadian database, comparison of the relative circadian amplitudes of nascent and mature transcripts, and regulation of circadian TFs are available in the Supplementary information.

## Additional Information

**Accession codes**: RNA-seq data of miR-378 over-expression and liver-specific *Bmal1* cKO were submitted to GEO under accession numbers GSE73308 and GSE73271.

**How to cite this article**: Wang, H. *et al.* Oscillating primary transcripts harbor miRNAs with circadian functions. *Sci. Rep.* **6**, 21598; doi: 10.1038/srep21598 (2016).

## Supplementary Material

Supplementary Tables

Supplementary Information

## Figures and Tables

**Table 1:** List of circadian oscillating miRNA primary transcripts sorted according to their circadian phases in circadian time (CT).

Primary miRNA transcript	Significance score	Circadian phase (CT)
mmu-mir-6935	11.33	0.33
mmu-mir-340	14.42	0.42
mmu-mir-190a	10.31	0.50
mmu-mir-7223	9.78	2.67
mmu-mir-5104	17.86	3.17
mmu-mir-6371	9.15	3.50
mmu-mir-1933	9.05	3.83
mmu-mir-3073a	9.40	4.00
mmu-mir-7036b	13.08	4.17
mmu-mir-1962	9.31	4.50
mmu-mir-1927	10.31	4.83
mmu-mir-2139	14.53	5.00
mmu-mir-7664	11.05	5.67
mmu-mir-1249	9.18	6.00
mmu-mir-5120	12.53	6.00
mmu-mir-1950	10.74	7.17
mmu-mir-7072	10.26	7.17
mmu-mir-101b	13.80	7.67
mmu-mir-1892	10.97	8.50
mmu-mir-7046	10.92	9.00
mmu-mir-378	9.25	9.17
mmu-mir-342	10.34	9.83
mmu-mir-29b-1~29a	16.18	10.00
mmu-mir-1190	9.64	10.33
mmu-mir-7048	13.09	10.33
mmu-mir-101a	11.64	10.50
mmu-mir-6896	15.58	11.33
mmu-mir-6908	12.16	11.83
mmu-mir-7661	13.57	12.00
mmu-mir-8114	10.05	12.00
mmu-mir-1947	13.31	12.67
mmu-mir-6953	9.63	12.83
mmu-mir-23b~27b~24-1	9.52	13.00
mmu-mir-1906-1	9.32	13.17
mmu-mir-6972	13.48	13.50
mmu-mir-6930	9.57	13.67
mmu-mir-455	10.44	14.00
mmu-mir-6971	9.52	14.50
mmu-mir-21a	10.18	16.33
mmu-mir-1191b	9.11	17.00
mmu-mir-484	10.18	17.00
mmu-mir-7687	9.46	17.17
mmu-mir-1936	12.34	19.67
mmu-mir-6353	10.62	19.67
mmu-mir-8107	10.06	20.00
mmu-mir-7063	10.25	20.33
mmu-mir-3087	13.90	20.50
mmu-mir-7022	11.37	20.50
mmu-mir-8093	9.64	20.50
mmu-mir-7052	10.12	20.67
mmu-mir-1955	11.44	20.83
mmu-mir-5131	13.49	21.17
mmu-mir-122	9.20	22.25
mmu-mir-130a	9.63	22.50
mmu-mir-6963	12.34	22.83
mmu-mir-3962	25.12	23.83
mmu-mir-6964	11.93	23.83

The significance score is defined as summed − (log2-transformed cosine fitting p-value).
