# Supplementary material for: Oscillating primary transcripts harbor miRNAs with circadian functions
Source: Sci Rep. 2016 Feb 22;6:21598. doi: 10.1038/srep21598 (PMC4761921; doi:10.1038/srep21598)
Supplement: Supplementary Information [file srep21598-s2.pdf]

Supplementary Information

Oscillating primary transcripts harbor miRNAs with circadian functions

Haifang Wang[1,2], Zenghua Fan[1,5], Meng Zhao[1,2], Juan Li[1], Minghua Lu[3], Wei Liu[4], Hao Ying[4], Mofang Liu[3], Jun Yan[1,2*]

[1]CAS-MPG Partner Institute for Computational Biology,

[2]Institute of Neuroscience, CAS Center for Excellence in Brain Science and Intelligence Technology,

[3]Institute for Biochemistry and Cell Biology,

[4]Institute for Nutritional Sciences,

Shanghai Institutes for Biological Sciences, Chinese Academy of Sciences, Shanghai 200031, China

[5]University of Chinese Academy of Sciences, Shanghai 200031, China

Supplementary Information

Supplementary Table of Contents

I. **Extended Experimental Procedures**

II. **Supplementary References**

III. **Supplementary Figures**

IV. **Supplementary Tables**

Table S1. miRNA primary transcripts identified by Vespucci using GRO-seq data. The genomic locations are based on mm9 assembly.

Table S2. Compare our miRNA primary annotation with the annotation from Saini et al.'s paper. The genomic locations are based on mm9 assembly.

Table S3. Primer sequences used for miRNA primary transcripts validation.

Table S4. All the collected circadian regulators ChIP-seq data for promoter analysis.

Table S5. Circadian regulators of oscillating miRNA primary transcripts. The numbers of datasets supporting the binding of regulators on each miRNA primary transcript are listed.

Table S6. Primer sequences used in BMAL1 ChIP-PCR experiment.

Table S7. Primer sequences used in miRNA qPCR experiment.

Table S8. ANOVA analysis of the miR-378 over-expression RNA-seq data. The RPKM and ANOVA p-values for all the UCSC genes were listed

Table S9. Functional annotation of miR-378 affected circadian genes. DAVID program were applied to identify the enriched biological functions for each group of genes using all the mouse genes as the background. Terms with p-value<0.05 were selected and redundant terms based on the clustering result from DAVID were removed.

Table S10. Primer sequences for cell cycle genes.

I. Extended Experimental Procedures

BMAL1 ChIP-PCR analysis

The liver samples from three mice were collected at ZT8. The liver tissues were first transferred into a Dounce homogenizer with cross-linking solution, then homogenized, and incubated for 10 min with gentle shaking. 0.125M final concentration of glycine was added to stop the fixation. The cross-linked cells were centrifuged at 4100 rpm for 5 min to remove the supernatant. The cells were lysed by incubation on ice for 10 min and the lysate was transferred to Bioruptor tubes with ChIP dilution buffer containing protease and histone deacetylase inhibitors, followed by sonication for 25 min. The lysate was purified by centrifugation at 13000 rpm for 10 min. Quant-iT was used to measure the amount of chromatin DNA. 4 μg of chromatin DNA was then incubated at 4°C over night on the rotating wheel with 2 μg of BMAL1 antibody (SANTA CRUZ, product ID:SC-8550) dissolving with ChIP buffer into 500 μl. The top 90% of cleared chromatin was washed by protein A/G Bynabeads. Beads were then washed two times with low-salt wash buffer, lithium chloride wash buffer, and TF buffer. 100μl 10% Chelex 100 slurry were added to the washed beads

and boiled for 10 min. Co-immunoprecipitated DNA fragments were incubated for 30 min at 55°C with Proteinase K and then the solution was centrifuged at 14000 rpm for 1 min at 4°C. For qPCR quantification, the equivalent of 5 μl of chromatin of each reaction was used in a 20-μl reaction using the primers listed in Supplementary Table S6. The input chromatin DNA from each sample was used as control.

Analysis of mouse liver circadian miRNA-seq data

The raw miRNA sequence reads were downloaded from Vollmers et al.'s FTP site[1]. In their experiment, two mouse liver samples were collected at CT21, CT25, CT29, CT33, CT37, CT41 and CT45 respectively. The 3' adaptors were trimmed by fastx_clipper program in Fastx_bin package (fastx_clipper -Q33 -a TGGAATTCTCGGGTGCCAAGGAACTCCAGTCAC -l 15 -M 10). Then the sequences were mapped to the mouse mm9 assembly by bowtie2 program (-N 0 -L 15). The read counts across samples for each mature miRNA were calculated by bedtools coverage and then normalized by the total read number in each sample. Cosine fitting p-value<0.05 and ANOVA p-value<0.05 as described in our previous paper[2] were used as cutoff to define the circadian oscillating miRNAs.

qPCR of mature miR-378-3p and miR-378-5p expression

For miRNA qPCR, we used the reverse-transcription primer with a target-specific stem-loop structure to overcome the problem in miRNA quantitation. First cDNA is reverse transcribed from total RNA samples using specific primer with PrimeScript™ RT reagent Kit (TAKARA)

according to the instruction. Then qPCR was performed with SYBR® Premix Ex Taq™ II (TAKARA) in three replicates in 20μl volumes. The real-time reaction mix consisted of 0.2μl RT product, 10μl of 2×SYBR Green PCR Master Mix, 0.4μl ROX Reference Dye II and 0.8μl samll RNA specific primer (10μM). qPCR assays were conducted on Applied Biosystems 7500 fast qPCR system (ABI). U6 RNA was used as the endogenous control. The primers for RT and PCR are listed in Supplementary Table S7.

Circadian miRNA target identification

The miRNA targets based on prediction and AGO CLIP-sequencing (CLIP-seq) were downloaded from StarBase (http://starbase.sysu.edu.cn/). The miRNA-target interactions from human and mouse were identified based on both predicted binding sites and AGO CLIP-Seq experimental evidence.

RNA-sequencing

Total RNA was extracted from each liver sample using Trizol. The RNA samples of Ad-378 and Ad-null mice were converted into Illumina sequencing libraries by using Illumina TruSeq RNA Sample Prep Kit v2 as per manufacturer's protocol, The RNA samples of *Bmal* cKO and wild-type mice were converted into Illumina sequencing libraries by using Illumina TruSeq Stranded mRNA Sample Preparation Kits V2 as per manufacturer's protocol which generates strand-specific RNA-seq library. Libraries of miR-378 over-expression samples and *Bmal* cKO samples were sequenced in two separate Illumina HiSeq 2000 lanes by using the 100-bp single strand chemistry.

RNA-seq data analysis

Fastq files containing raw sequence reads were aligned to mouse genome (mm9) by Tophat program (default parameters)[3]. Then bedtools (v2.16.2) were applied to calculate the number of reads of each transcript based on the gene annotations from UCSC genome browser. The transcripts with summed reads number<20 in the samples were removed. The reads numbers were normalized to RPKM. Two way ANOVA with treatment (Ad-378 vs. Ad-null, control vs. BMAL1 cKO) and time (CT10 vs. CT22 in miR-378 over-expression, CT0 vs. CT12 in *Bmal1* KO) as two factors were applied to identify the differentially expressed genes. The RPKM and ANOVA p-values for all the UCSC genes were listed in Table S8.

miR-378-3p targets in NIH-3T3 cell

Genome-wide study of miR-378-3p targets in NIH-3T3 cells were downloaded from GEO database (GSE34873). Robust Multichip Average (RMA) normalization was applied to normalize the raw data. One-way ANOVA and Tukey's test were applied to obtain the differentially expressed genes between control and miR-378-3p inhibition and between control and miR-378 mimic. The genes with ANOVA p-value<0.05, over-expressed in miR-378-3p inhibition and under-expressed in miR-378-3p mimic with log2-transformed fold change>0.3 were defined as miR-378-3p targets.

Mouse liver circadian database

Six mouse liver circadian microarray datasets were collected. Five of them have been described and integrated in our pervious paper[2], and the other dataset from Hughes et al.

paper[4] were analyzed as previous described[2] . Mouse liver circadian genes were defined as oscillating in two out of six mouse liver circadian datasets. The mean circadian phase across the six datasets was calculated using circular (R package) and defined as the circadian phase of the mouse liver circadian oscillating genes.

Comparison of the relative circadian amplitudes of nascent and mature transcripts

Sequencing reads of RNA-seq and Nascent-seq[5] were downloaded from GEO database (GSE36916) and mapped to mouse genome (mm9) by Bowtie2 program (default parameters)[6]. The read number of each transcript (primary transcripts were based on the annotation from Vespucci program and mature transcript were based on the annotation from UCSC (mm9)) was normalized to RPKM. The circadian peak and trough of each transcript were calculated by fitting them to cosine functions with 24 hours' period and shifting phases as described previously[2]. The peak to trough ratio was defined as relative circadian amplitude.

Regulation of circadian TFs

Two types of data were used to define the target of BMAL1/CLOCK and REV-ERBα/β□in mouse liver: physical binding data (ChIP-seq) and functional data (TF knock-out microarray or RNA-seq). ChIP-seq data and methods to define the TF binding sites have been described in the previous section. The normalized microarray data for liver specific *Rer-erbα/β* double cKO (Cho et al. 2012) were downloaded from GEO database (GSE34020). Two-way ANOVA with circadian time and genotype as two factors was applied to both *Bmal1* and *Rer-erbα/β* cKO data to obtain the differentially expressed genes and the TF target circadian genes were

defined by ANOVA p-value for genotype<0.05 and ANOVA p-value for circadian time<0.05.

II. Supplementary References

1. Vollmers, C. *et al.* Circadian oscillations of protein-coding and regulatory RNAs in a highly dynamic mammalian liver epigenome. *Cell Metab.* **16**, 833-45 (2012).
2. Yan, J., Wang, H., Liu, Y. & Shao, C. Analysis of gene regulatory networks in the mammalian circadian rhythm. *PLoS Comput. Biol.* **4**, e1000193 (2008).
3. Kim, D. *et al.* TopHat2: accurate alignment of transcriptomes in the presence of insertions, deletions and gene fusions. *Genome Biol.* **14**, R36 (2013).
4. Hughes, M.E. *et al.* Harmonics of circadian gene transcription in mammals. *PLoS Genet.* **5**, e1000442 (2009).
5. Menet, J.S., Rodriguez, J., Abruzzi, K.C. & Rosbash, M. Nascent-Seq reveals novel features of mouse circadian transcriptional regulation. *Elife* **1**, e00011 (2012).
6. Langmead, B. & Salzberg, S.L. Fast gapped-read alignment with Bowtie 2. *Nat. Methods* **9**, 357-9 (2012).

III. Supplementary Figures

Figure S1. Genome browser view of pri-mir-29a~29b-1 to illustrate how we define the miRNA primary transcript and the regulators of the primary transcript. GRO-seq were used to define the primary transcript, promoter marker's ChIP-seq (H3K4me3 and polⅡ) were used to define the 5' end of the primary transcript, and ChIP-seq were used to identify the regulators of miRNA primary transcripts.

Figure S2. Expression pattern of miR-26b-5p and miR-150-5p in mouse liver from Vollmers et al.'s miRNA-seq data. The x-axis represents the circadian time (CT), while the y-axis represents the relative expression values.

Figure S3. miR-378 over-expression efficiency. *** represents t-test (unpaired, two-side) p-value<0.001.

Figure S4. Expression pattern of core circadian genes in ad-378 and Ad-null mice. ** represents ANOVA p-value for genotype<0.01.

Figure S5. qPCR analysis of the circadian oscillating cell cycle genes upon miR-378 over-expression and illustration of their putative miR-378 binding site. (a) qPCR analysis ofr the circadian oscillating cell cycle genes. ** represents ANOVA p-value for genotype<0.01, * represents ANOVA p-value for genotype<0.05, # represents ANOVA p-value for genotype<0.1. (b) The alignmenta of miR-378 with its putative binding sites.

Figure S6. The expression patterns of the three genes involved in transcriptional regulation (*Fus,Rdbp*, and *Pnrc2*) that are under rhythmic post-transcriptional regulation through miR-378. The left figures show the expression patterns of nascent and

mature transcripts, while the right figures show the expression patterns upon miR-378 over-expression. ** represents ANOVA p-value for genotype<0.01, * represents ANOVA p-value for genotype<0.05.

Figure S7. miR-378 mediates the circadian control of cell cycle and metabolism by cooperating with other circadian TFs. Regulatory network formed by miR-378 and other circadian TFs (BMAL/CLOCK, REV-ERBα/β). Red lines represent activation, while blue lines represent repress. The green nodes represent peak around CT10, while the purple nodes represent peak around CT22.

Figure S1

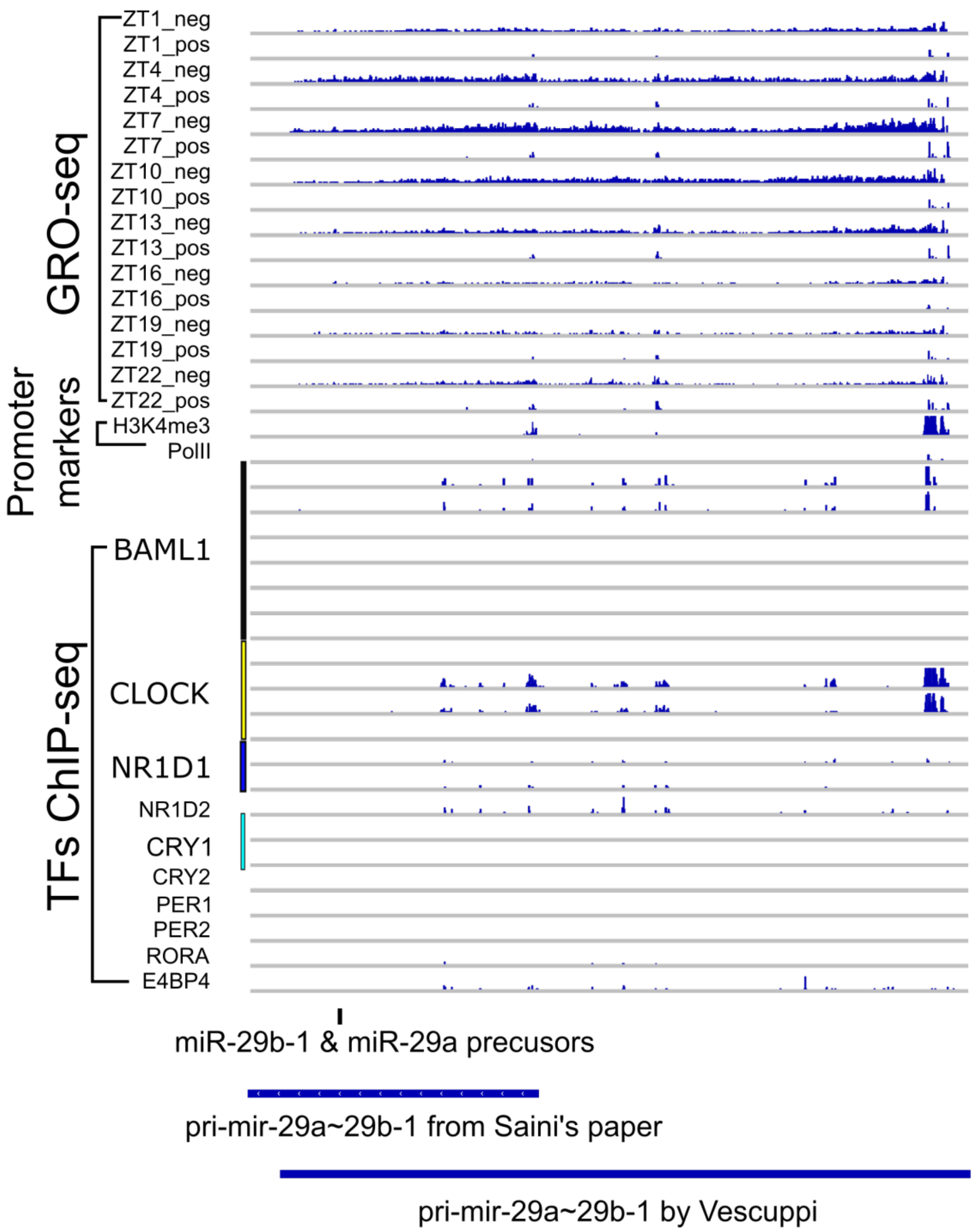

GRO-seq
ZT1_neg
ZT1_pos
ZT4_neg
ZT4_pos
ZT7_neg
ZT7_pos
ZT10_neg
ZT10_pos
ZT13_neg
ZT13_pos
ZT16_neg
ZT16_pos
ZT19_neg
ZT19_pos
ZT22_neg
ZT22_pos
Promoter markers
H3K4me3
PolII
TFs ChIP-seq
BAML1
CLOCK
NR1D1
NR1D2
CRY1
CRY2
PER1
PER2
RORA
E4BP4
miR-29b-1 & miR-29a precusors
pri-mir-29a~29b-1 from Saini's paper
pri-mir-29a~29b-1 by Vescuppi

Figure S2

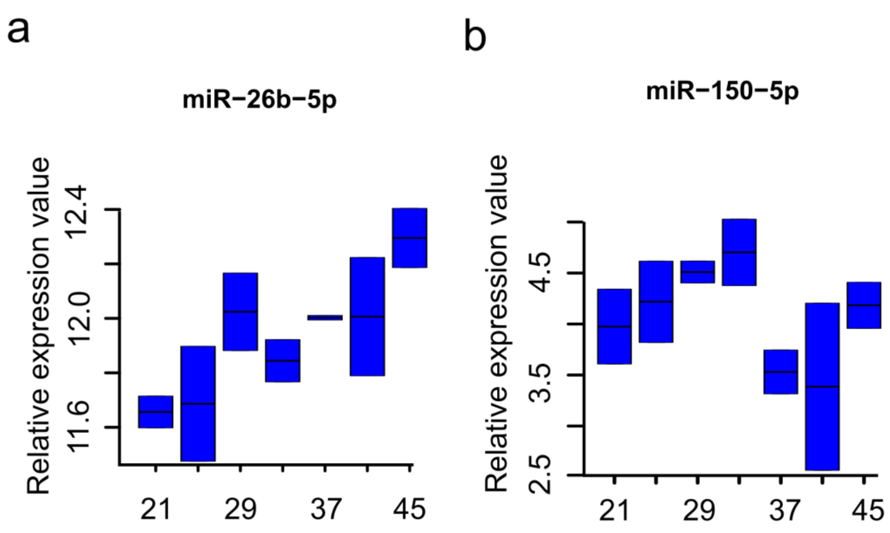
a
miR−26b−5p
Relative expression value
11.6
12.0
12.4
21
29
37
45
b
miR−150−5p
Relative expression value
2.5
3.5
4.5
21
29
37
45

Figure S3

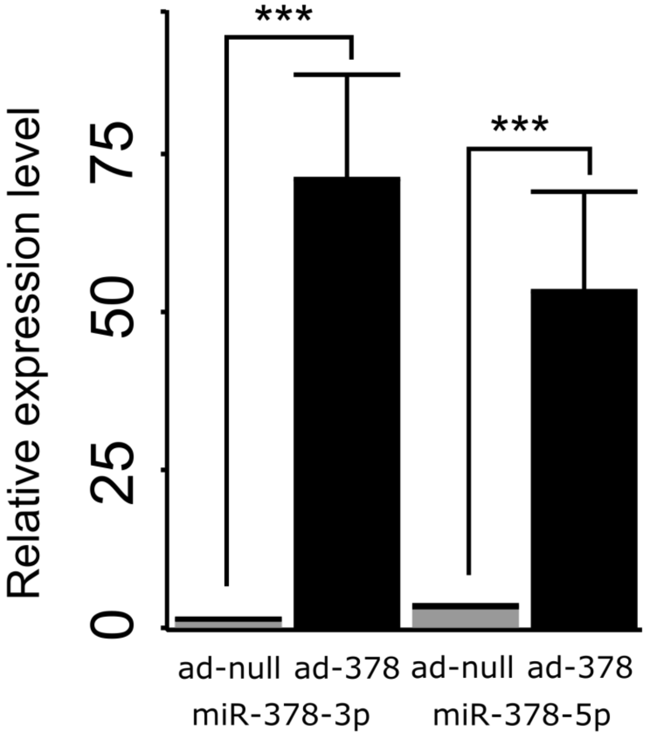
***
***
Relative expression level
0
25
50
75
ad-null
ad-378
ad-null
ad-378
miR-378-3p
miR-378-5p

Figure S4

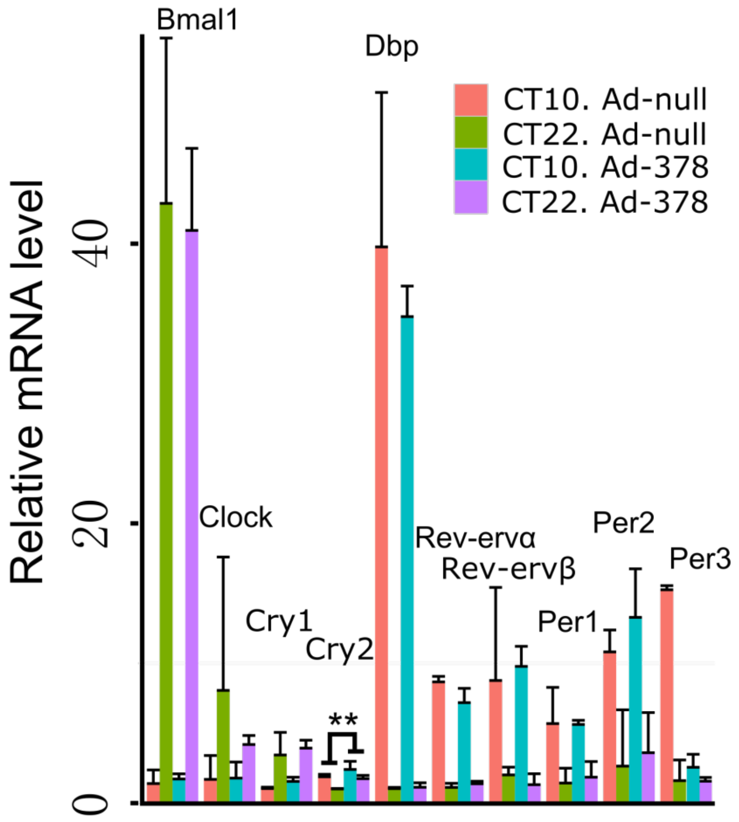

Relative mRNA level
0
20
40
Bmal1
Clock
Cry1
Cry2
**
Dbp
Rev-ervα
Rev-ervβ
Per1
Per2
Per3
CT10. Ad-null
CT22. Ad-null
CT10. Ad-378
CT22. Ad-378

Figure S5

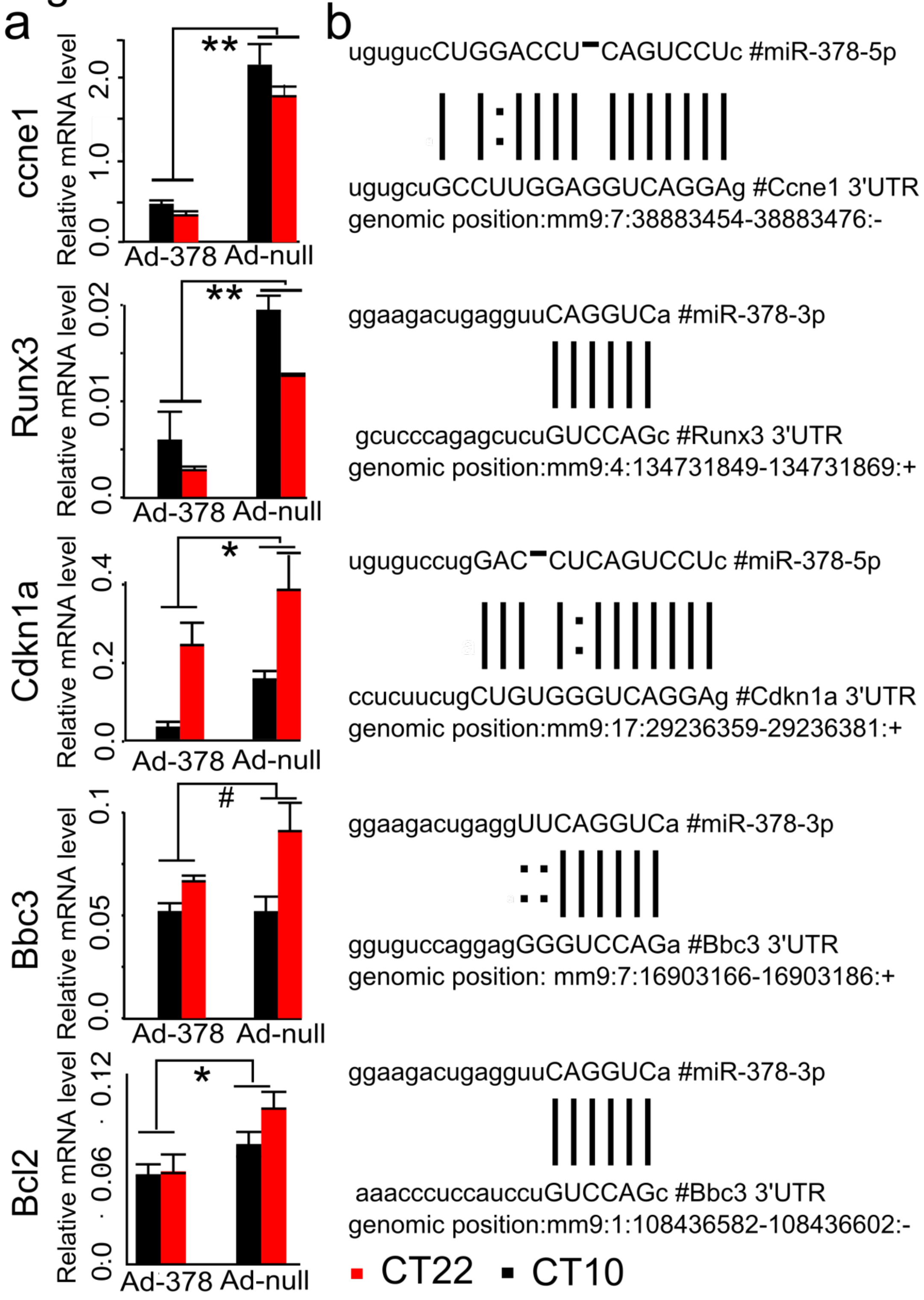
a
b
ccne1
Runx3
Cdkn1a
Bbc3
Bcl2
Relative mRNA level
Ad-378 Ad-null
**
*
#
ugugucCUGGACCU-CAGUCCUc #miR-378-5p
ugugcuGCCUUGGAGGUCAGGAg #Ccne1 3'UTR
genomic position:mm9:7:38883454-38883476:-
ggaagacugagguuCAGGUCa #miR-378-3p
gcucccagagcucuGUCCAGc #Runx3 3'UTR
genomic position:mm9:4:134731849-134731869:+
uguguccugGAC-CUCAGUCCUc #miR-378-5p
ccucuucugCUGUGGGUCAGGAg #Cdkn1a 3'UTR
genomic position:mm9:17:29236359-29236381:+
ggaagacugaggUUCAGGUCa #miR-378-3p
gguguccaggagGGGUCCAGa #Bbc3 3'UTR
genomic position: mm9:7:16903166-16903186:+
ggaagacugagguuCAGGUCa #miR-378-3p
aaacccuccauccuGUCCAGc #Bbc3 3'UTR
genomic position:mm9:1:108436582-108436602:-
CT22
CT10

Figure S6

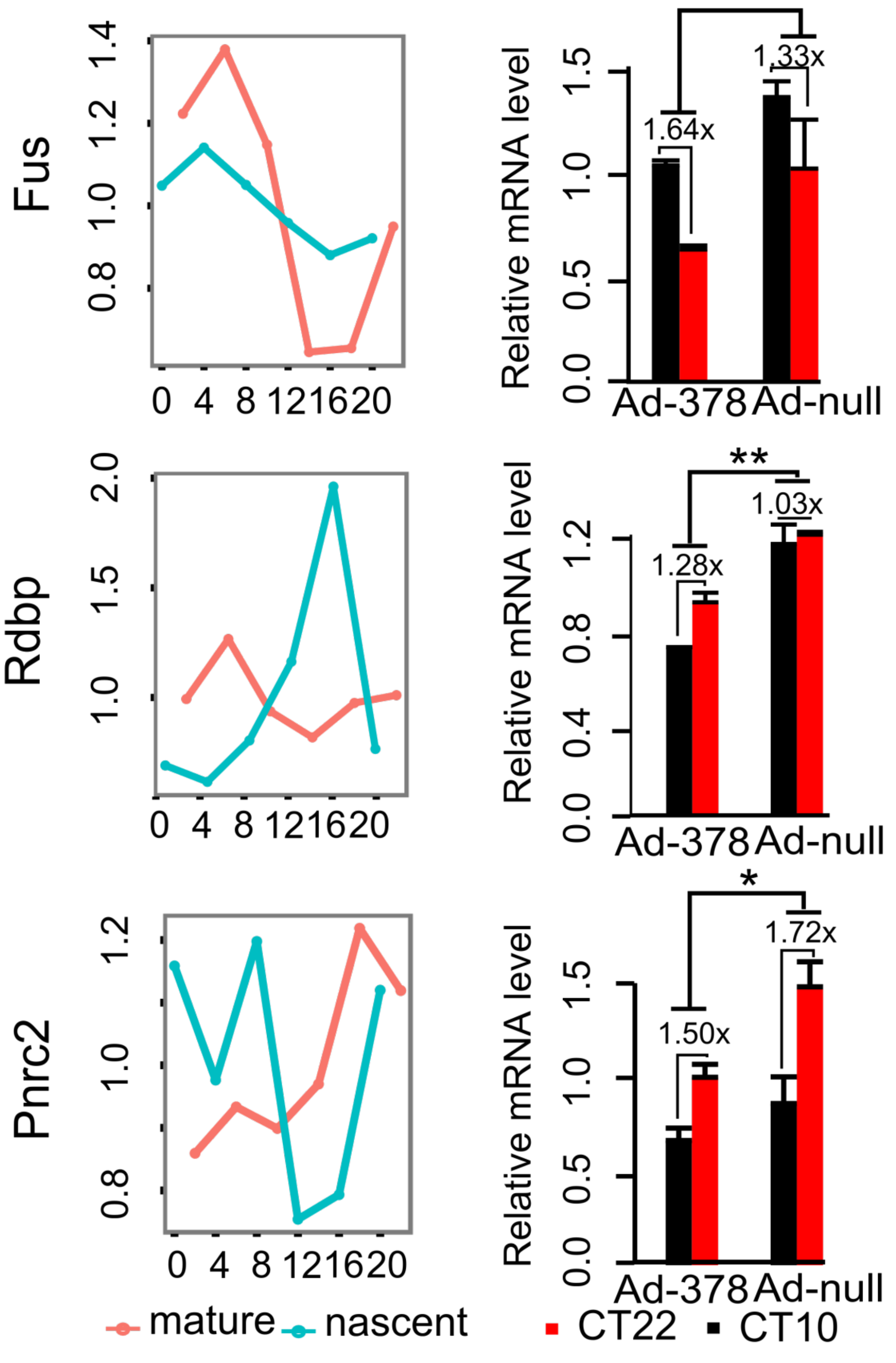

Fus
Rdbp
Pnrc2
0.8
1.0
1.2
1.4
2.0
1.5
0 4 8 12 16 20
Relative mRNA level
0.0
0.5
0.4
0.8
1.2
*
**
1.64x
1.33x
1.28x
1.03x
1.50x
1.72x
Ad-378
Ad-null
mature
nascent
CT22
CT10

Figure S7

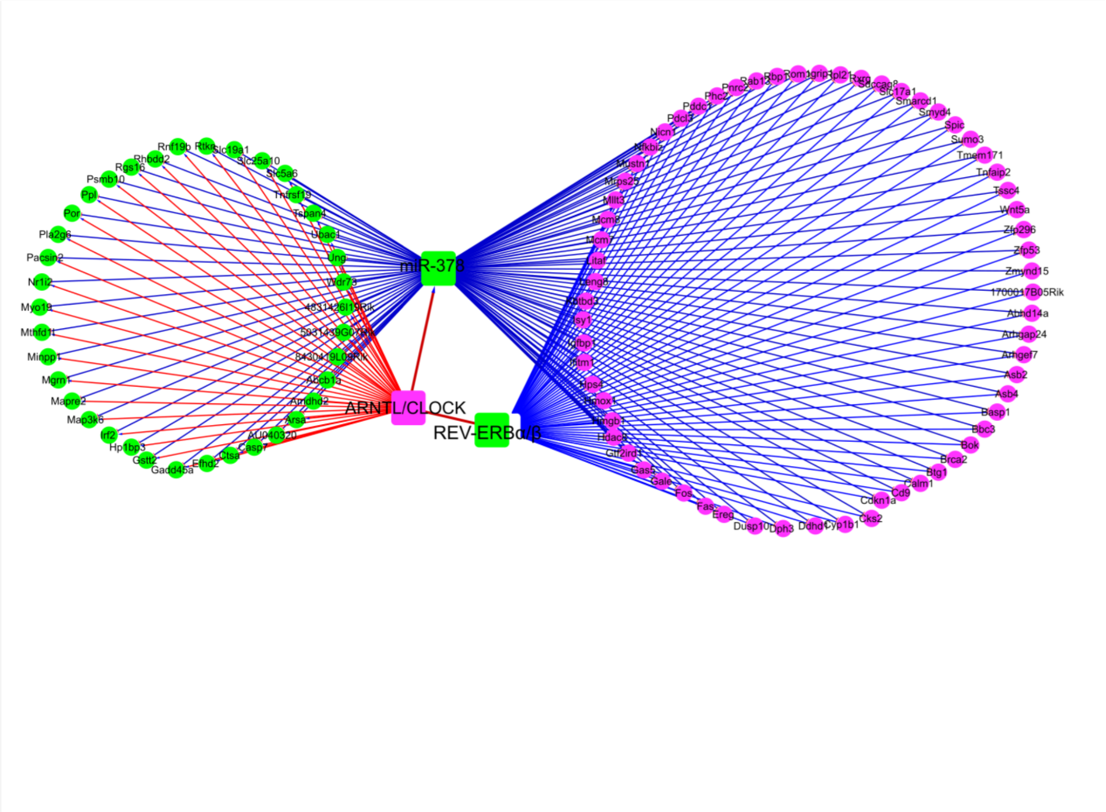

miR-378
ARNTL/CLOCK
REV-ERBα/β
Rnf19b
Rtkn
Slc19a1
Slc25a10
Slc5a6
Tnfrsf19
Tspan4
Ubac1
Ung
Wdr73
4831426I19Rik
5031439G07Rik
8430419L09Rik
Abcb1a
Amdhd2
Arsa
AU040320
Casp7
Ctsa
Efhd2
Gadd45a
Gstt2
Hp1bp3
Irf2
Map3k6
Mapre2
Mgrn1
Minpp1
Mthfd1l
Myo19
Nr1i2
Pacsin2
Pla2g6
Por
Ppl
Psmb10
Rgs16
Rhbdd2
Rab13
Rbp1
Rpl21
Rxrg
Sdccag8
Slc17a1
Smarcd1
Smyd4
Spic
Sumo3
Tmem171
Tnfaip2
Tssc4
Wnt5a
Zfp296
Zfp53
Zmynd15
1700017B05Rik
Abhd14a
Arhgap24
Arhgef7
Asb2
Asb4
Basp1
Bbc3
Bok
Brca2
Btg1
Calm1
Cd9
Cdkn1a
Cks2
Cyp1b1
Dph3
Dusp10
Ereg
Fas
Fos
Gale
Gas5
Gtf2ird1
Hdac8
Hmgb1
Hmox1
Hps4
Ifitm1
Igfbp1
Isy1
Kbtbd3
Leng8
Litaf
Mcm7
Mcm8
Mllt3
Mrps25
Mustn1
Nfkbiz
Nicn1
Pdcl3
Pddc1
Phc2
Pnrc2

IV. Supplementary Tables

Table S2. Compare our miRNA primary annotation with the annotation from Saini et al.'s paper.

Chr	Start from Saini et al.	End from Saini et al.	Primary miRNA	Start from ours	End from ours
chr13	48630000	48643000	let-7a-1~7f-1~7d	48612083	48642477
chr15	68158977	68195000	mir-30d~30b	68145723	68194788
chr17	17963852	17968000	mir-99b~let-7e~mir-125a	17967147	17976084
chr1	196863023	196865102	mir-29b-2~29c	196843592	196864148
chr6	30972660	31063672	mir-29b-1~29a	30998789	31171790
chr4	101019136	101028814	mir-101a	101019126	101028885
chr3	28981483	29415524	mmu-mir-551b	28981448	29438575
chr14	62245717	62300947	mir-15a~16-1	62221667	62301253
chr16	43639978	43642390	mmu-mir-568	43619287	43649451

Table S3. Primer sequences used for miRNA primary transcripts validation.

Primary miRNA	Formard	Reverse
pri-mir-mir122	CAACACAGGGCAAAGACAGC	AGAGGGGCTGAGGATGCTAA
pri-mir-101a	CGCTCCTGTGTTCACCACTCTTC	TTGTCCTCAGCATAACCGTCTTCAT
pri-mir-23b~27b~24-1	GCCACTGAATACTGTCATCGTC	GCATCCCAGATAACACGGAGG
pri-mir-29a~29b-1	CATGGAACAGATTAACCGCACT	CATTGAGCTGTCTCCCCGGTA
pri-mir-340	CCGTGGCTCTCTAGTCTTCGT	TGGTTCCTGTCCCGAGCAT
pri-mir-mir378	GGAGCCAAGGTTGAGCAAGGT	AATGAGACTGCGAAGCCAAGGT

Table S4. All the collected circadian TFs ChIP-seq data for promoter analysis.

GSE ID	TFs	Reference(PMID)
GSE36874	BMAL1 & CLOCK	23150795
GSE53828	BMAL1 & CLOCK & CRY1	24385426
GSE39977	BMAL1 & CLOCK & PER1 & PER2 & CRY1 & CRY2	22936566
GSE26602	BMAL1	21364973
GSE34019	NR1D1 & NR1D2	22460952
GSE36375	NR1D1 & NR1D2	22474260 & 21393543
GSE59486	E4BP4 & RORA	25416951

Table S5. Circadian regulators of oscillating miRNA primary transcripts.

	BMAL1	CLOCK	CRY1	CRY2	E4BP4	PER1	PER2	REV-ERBα	REV-ERBβ	RORA
pri-mir-23b~27b~24-1	2	2	1	0	1	0	0	2	1	1
pri-mir-29b-1~29a	6	3	1	1	1	1	1	2	1	1
pri-mir-101a	4	3	1	1	1	1	1	2	1	1
pri-mir-130a	1	2	1	1	1	0	1	2	1	0
pri-mir-190a	2	1	0	1	1	1	1	2	1	1
pri-mir-122	2	3	1	1	1	0	1	2	1	1
pri-mir-21a	1	1	1	1	1	0	0	2	1	1
pri-mir-340	4	3	1	1	1	1	1	2	1	1
pri-mir-342	2	1	1	1	0	1	0	0	0	1
pri-mir-101b	5	3	1	1	1	1	1	2	1	1
pri-mir-378a	7	3	2	1	1	1	1	2	1	1
pri-mir-484	1	2	0	0	1	0	1	0	1	0
pri-mir-1249	3	1	1	1	1	1	1	2	1	0
pri-mir-455	4	3	2	1	1	1	1	2	1	1
pri-mir-1190	3	3	1	1	1	1	1	2	1	1
pri-mir-1892	2	2	1	1	0	0	0	0	0	0
pri-mir-1906-1	2	2	2	1	1	0	1	2	1	1
pri-mir-1927	5	3	2	1	1	0	1	2	1	1
pri-mir-1933	2	3	1	1	1	0	1	1	1	1
pri-mir-1936	2	2	1	1	1	1	1	2	1	1
pri-mir-1947	3	2	1	0	1	0	0	2	1	0
pri-mir-1950	1	2	1	0	0	0	0	0	0	1
pri-mir-1955	2	2	2	1	1	0	0	1	0	0
pri-mir-1962	3	3	1	1	1	1	0	1	1	1
pri-mir-2139	5	3	1	1	1	1	1	2	1	1
pri-mir-3073a	4	3	2	1	1	1	1	2	1	1
pri-mir-3087	1	2	0	0	0	0	0	0	0	0
pri-mir-3962	1	0	0	0	0	0	0	0	0	0
pri-mir-5104	3	2	1	1	1	1	1	2	1	1
pri-mir-5120	3	3	2	1	1	0	0	1	0	1
pri-mir-5131	2	2	0	1	1	1	1	2	1	1
pri-mir-6353	3	3	2	1	1	1	1	2	1	1
pri-mir-6371	2	3	1	1	1	1	1	1	1	1
pri-mir-6896	4	2	1	1	1	0	1	2	1	1
pri-mir-6908	6	3	1	1	1	1	1	1	1	1
pri-mir-6930	5	3	1	1	1	1	1	2	1	1
pri-mir-6935	3	2	1	1	0	0	1	2	0	0
pri-mir-6953	3	2	1	1	0	1	1	0	0	1
pri-mir-6963	0	2	0	1	1	0	0	1	0	1

pri-mir-6964	3	3	1	1	1	0	1	2	1	1
pri-mir-6972	4	3	2	1	1	1	1	2	1	1
pri-mir-7022	2	3	1	1	1	0	0	2	1	0
pri-mir-7046	2	2	0	0	0	0	1	0	1	1
pri-mir-7048	6	3	1	1	1	1	1	2	0	0
pri-mir-7052	2	2	1	1	1	0	0	1	0	1
pri-mir-7063	2	3	1	1	1	0	1	2	1	1
pri-mir-7072	0	1	0	0	0	0	0	0	0	0
pri-mir-7223	4	2	2	1	1	0	1	2	1	1
pri-mir-7036b	0	2	1	0	0	0	0	0	0	0
pri-mir-7661	7	3	2	1	1	1	1	2	1	1
pri-mir-7664	3	3	2	1	1	1	1	2	1	1
pri-mir-1191b	3	3	1	1	1	1	1	2	1	1
pri-mir-7687	7	3	1	1	1	1	1	2	1	1
pri-mir-8093	3	2	1	1	0	0	1	0	0	0
pri-mir-8107	4	3	1	1	1	1	1	1	0	1
pri-mir-8114	2	2	1	1	0	0	1	1	0	0

Table S6. Primer sequences used in BMAL1 ChIP-PCR experiment.

miRNAs/controls	forward primers	reverse primers
miR-24	GGGGTCTTAGGGATGCACTT	CCTGGGCACATGTGTTCAC
miR-101a	CTTGTGCTCCATCCTTCTGC	AGAAAATGCAGCGGTCTTCC
miR-378	AGCTAGCTACTCACCGCCTA	GTGCTACCACGTGTCTGTGA
Dbp (positive control)	ACACAAGTTCAGCCCCTCAC	GGCAAGAACCAATCACGTCT
Gapdh (negative control)	AACTTTGGCATTGTGGAAGG	ACACATTGGGGGTAGGAACA

Table S7. Primer sequences used in miRNA qPCR experiment.

<table>
<tr><th>miRNAs</th><th>RT primers</th><th>Forward primers</th><th>Universal reverse primer</th></tr>
<tr><td>miR-378-3p</td><td>TCAACTGGTGTCGTGGAGTCGGC AATTCAGTTGAGccttctga</td><td>ACACTCCAGCTGGGACT GGACTTGGAGTCA</td><td rowspan="3">CTCAACTGGTGTCG TGGAGTCGG</td></tr>
<tr><td>miR-378-5p</td><td>TCAACTGGTGTCGTGGAGTCGGC AATTCAGTTGAGacacagga</td><td>ACACTCCAGCTGGGCTC CTGACTCCAGGTC</td></tr>
<tr><td>u6</td><td>CTCAACTGGTGTCGTGGAGTCGG CAATTCAGTTGAGAAAAATAT</td><td>ACACTCCAGCTGGGCGC AAATTCGTGAAGC</td></tr>
</table>

Table S9. Functional annotation of miR-378 affected circadian genes.

Groups	Terms	P-value
Group Ⅰ	GO:0007049~cell cycle	2.00E-04
	GO:0055085~transmembrane transport	1.05E-03
	GO:0046651~lymphocyte proliferation	1.06E-03
	GO:0010605~negative regulation of macromolecule metabolic process	3.12E-03
	GO:0016481~negative regulation of transcription	4.42E-03
	GO:0051480~cytosolic calcium ion homeostasis	5.34E-03
	GO:0001775~cell activation	6.62E-03
	GO:0007040~lysosome organization	7.64E-03
	GO:0016265~death	8.85E-03
Group Ⅱ	GO:0007599~hemostasis	1.52E-06
	GO:0009611~response to wounding	1.85E-03
	GO:0044242~cellular lipid catabolic process	2.87E-03
	GO:0046907~intracellular transport	6.11E-03
	GO:0055114~oxidation reduction	6.68E-03
	GO:0019725~cellular homeostasis	7.81E-03
	GO:0055085~transmembrane transport	8.61E-03
	GO:0006508~proteolysis	1.45E-02
	GO:0032101~regulation of response to external stimulus	1.54E-02
Group Ⅲ	GO:0006412~translation	1.13E-05
	GO:0045449~regulation of transcription	6.72E-04
	GO:0043068~positive regulation of programmed cell death	8.26E-04
	GO:0051051~negative regulation of transport	3.40E-03
	GO:0008360~regulation of cell shape	4.21E-03
	GO:0033554~cellular response to stress	4.70E-03
	GO:0006915~apoptosis	6.06E-03
	GO:0051048~negative regulation of secretion	6.24E-03
	GO:0051789~response to protein stimulus	7.59E-03
	GO:0007015~actin filament organization	7.86E-03
	GO:0042770~DNA damage response, signal transduction	8.37E-03
Group Ⅳ	GO:0050830~defense response to Gram-positive bacterium	4.31E-02

Table S10. Primer sequences for cell cycle genes.

Genes	Forward primers	Reverse primers
Ccne1	TGAGTTCCAAGCCCAAGTCC	TCTTGCAAAAACACGGCCAC
Runx3	AAGTGGGTCTGAACCCAACC	GCTCGGGTCTCGTATGAAGG
Cdkn1a	CTTGTCGCTGTCTTGCACTC	GGGCACTTCAGGGTTTTCTC
Bbc3	GTGTGGAGGAGGAGGAGTG	TGTCGATGCTGCTCTTCTTG
Bcl2	TCGCAGAGATGTCCAGTCAG	CACCCCATCCCTGAAGAGTT
Tbp (control)	GCAGCCTCAGTACAGCAATC	ACAGCCAAGATTCACGGTAGA
